# Histology and transcriptome insights into the early processes of intestinal anastomotic healing: a rat model

**DOI:** 10.1093/bjsopen/zrad099

**Published:** 2023-10-19

**Authors:** Claire P M van Helsdingen, Aurelia C L Wildeboer, Konstantina Zafeiropoulou, Audrey C H M Jongen, Joanna W A M Bosmans, Camille Gallé, Theodorus B M Hakvoort, Marion J J Gijbels, Wouter J de Jonge, Nicole D Bouvy, Andrew Y F Li Yim, Joep P M Derikx

**Affiliations:** Emma Children’s Hospital, Amsterdam UMC, location University of Amsterdam, Paediatric Surgery, Amsterdam, The Netherlands; Amsterdan UMC, location University of Amsterdam, Tytgat Institute for Liver and Intestinal Research, Amsterdam, The Netherlands; Amsterdam Gastroenterology Endocrinology Metabolism, Amsterdam, The Netherlands; Amsterdam Reproduction and Development, Amsterdam, The Netherlands; Emma Children’s Hospital, Amsterdam UMC, location University of Amsterdam, Paediatric Surgery, Amsterdam, The Netherlands; GROW, School for Oncology and Developmental Biology, Maastricht University, Maastricht, The Netherlands; Emma Children’s Hospital, Amsterdam UMC, location University of Amsterdam, Paediatric Surgery, Amsterdam, The Netherlands; Amsterdan UMC, location University of Amsterdam, Tytgat Institute for Liver and Intestinal Research, Amsterdam, The Netherlands; Amsterdam Gastroenterology Endocrinology Metabolism, Amsterdam, The Netherlands; Department of Surgery, Maastricht University Medical Center, Maastricht, The Netherlands; Department of Surgery, Maastricht University Medical Center, Maastricht, The Netherlands; Department of General Surgery, Maastricht University, Maastricht, The Netherlands; Amsterdan UMC, location University of Amsterdam, Tytgat Institute for Liver and Intestinal Research, Amsterdam, The Netherlands; Amsterdam Gastroenterology Endocrinology Metabolism, Amsterdam, The Netherlands; Department of Pathology, Maastricht University Medical Center, Maastricht, The Netherlands; NUTRIM School of Nutrition and Translational Research in Metabolism, Maastricht University Medical Center, Maastricht, The Netherlands; Department of Medical Biochemistry, Amsterdam UMC, location University of Amsterdam, Experimental Vascular Biology, Amsterdam, The Netherlands; Amsterdam Infection and Immunity, Amsterdam, The Netherlands; Amsterdam Cardiovascular Sciences, Amsterdam, The Netherlands; Amsterdan UMC, location University of Amsterdam, Tytgat Institute for Liver and Intestinal Research, Amsterdam, The Netherlands; Amsterdam Gastroenterology Endocrinology Metabolism, Amsterdam, The Netherlands; Department of Surgery, University of Bonn, Bonn, Germany; Department of Surgery, Maastricht University Medical Center, Maastricht, The Netherlands; NUTRIM School of Nutrition and Translational Research in Metabolism, Maastricht University Medical Center, Maastricht, The Netherlands; Emma Children’s Hospital, Amsterdam UMC, location University of Amsterdam, Paediatric Surgery, Amsterdam, The Netherlands; Amsterdan UMC, location University of Amsterdam, Tytgat Institute for Liver and Intestinal Research, Amsterdam, The Netherlands; Amsterdam Gastroenterology Endocrinology Metabolism, Amsterdam, The Netherlands; Amsterdam Reproduction and Development, Amsterdam, The Netherlands; Department of Human Genetics, Amsterdam UMC, location University of Amsterdam, Genome Diagnostics Laboratory, Amsterdam, The Netherlands; Emma Children’s Hospital, Amsterdam UMC, location University of Amsterdam, Paediatric Surgery, Amsterdam, The Netherlands; Amsterdam Gastroenterology Endocrinology Metabolism, Amsterdam, The Netherlands; Amsterdam Reproduction and Development, Amsterdam, The Netherlands

## Abstract

**Background:**

Understanding the early processes underlying intestinal anastomotic healing is crucial to comprehend the pathophysiology of anastomotic leakage. The aim of this study was to assess normal intestinal anastomotic healing and disturbed healing in rats to investigate morphological, cellular and intrinsic molecular changes in the anastomotic tissue.

**Method:**

Anastomoses were created in two groups of Wistar rats, using four sutures or 12 sutures to mimic anastomotic leakage and anastomotic healing respectively. At 6, 12, 24 hours and 2, 3, 5 and 7 days, anastomotic tissue was assessed macroscopically using the anastomotic complication score and histologically using the modified Ehrlich–Hunt score. Transcriptome analysis was performed to assess differences between anastomotic leakage and anastomotic healing at the first three time points to find affected genes and biological processes.

**Results:**

Ninety-eight rats were operated on (49 animals in the anastomotic leakage and 49 in the anastomotic healing group) and seven rats analysed at each time point. None of the animals with 12 sutures developed anastomotic leakage macroscopically, whereas 35 of the 49 animals with four sutures developed anastomotic leakage. Histological analysis showed increasing influx of inflammatory cells up to 3 days in anastomotic healing and up to 7 days in anastomotic leakage, and this increase was significantly higher in anastomotic leakage at 5 (*P* = 0.041) and 7 days (*P* = 0.003). Transcriptome analyses revealed large differences between anastomotic leakage and anastomotic healing at 6 and 24 hours, mainly driven by an overall downregulation of genes in anastomotic leakage.

**Conclusion:**

Transcriptomic analyses revealed large differences between normal and disturbed healing at 6 hours after surgery, which might eventually serve as early-onset biomarkers for anastomotic leakage.

## Teaser

In order to develop efficient interventions preventing anastomotic leakage and to better predict if a patient will develop anastomotic leakage, it is crucial to understand the underlying mechanisms of normal and disturbed anastomotic healing. In this animal model, the healing process over time was investigated and it was observed that the sequential phases of intestinal healing resemble the sequential phases of skin healing. Furthermore, 6 hours after anastomosis creation, transcriptional differences are present in the anastomotic tissue between healing and disturbed healing. These findings could be used as the first step to truly understand the disrupted healing processes underlying anastomotic leakage, which in turn might be used as potential early-onset biomarkers of anastomotic leakage.

## Introduction

Anastomotic leakage (AL) remains one of the most frequent complications in colorectal surgery with an incidence ranging between 3 and 30 per cent^1^. AL is typically diagnosed 5–8 days after surgery and is associated with severe complications, emergency interventions and an increased mortality risk^1,2^.

Over the last few decades, researchers have focused on preventing AL by investigating various surgery- and patient-related factors, which has guided optimization of surgical techniques and patient selection for de-functioning ostomy to protect the anastomosis. Despite these improvements, AL rates have remained stable. In addition, predicting surgical outcome remains challenging due to the heterogeneous clinical presentation with even the most optimized, young and healthy patients developing AL^3–5^. In part, this is due to the lack of understanding and monitoring of processes involved in anastomotic healing (AH). Ideally, we would be able to monitor the AH process in real time such that we could detect early derailment and consequently intervene to minimize severe complications^6,7^. Current knowledge on the mechanism of AH is largely derived from cutaneous wound healing, which has been thoroughly previously studied, as models of cutaneous wound healing are relatively simple to generate and visualize in real time^8^. Although the fundamentals of cutaneous healing might be roughly applicable to healing of intestinal anastomoses, a few differences, including collagen types, collagen metabolism and wound environment, exist between the two entities^2^.

While multiple studies have investigated AH by studying single components, such as the role of COX-2 and the mucus layer, little is known about the overall normal healing process and derangements of healing that may be the basis of AL^9,10^. In addition, there is a lack of insight into the sequential order in which the different processes take place over time. In most experimental studies, the animals are killed at only one time point between 3 and 10 days after creation of the anastomosis, which provides only a snapshot of the healing/leakage processes and does not provide insight into the dynamics of the ongoing processes^9–12^.

This study aimed to conducted a longitudinal analysis comparing histopathological evaluation and transcriptomic analyses during the first 24 hours to investigate the fundamental processes underlying intestinal anastomotic healing and leakage in animal models of normal/healthy anastomotic healing and impaired/pathologic healing.

## Methods

### Animals

Male Wistar rats, weighing 250–300 grams, were used (Harlan Laboratories, Boxmeer, The Netherlands). The animals were housed two/three in a cage with a 12/12 day/night cycle at the Central Animal Facility of Maastricht University. Food and water were provided *ad libitum*. All animal experiments were approved by the Maastricht University Animal Experiments Committee (DEC. nr. 2014-120) and the protocol complied with the Dutch Animal Experimental Act. The ARRIVE guidelines 2.0 for reporting animal research were followed^13^.

### Study design

**Fig. 1:**
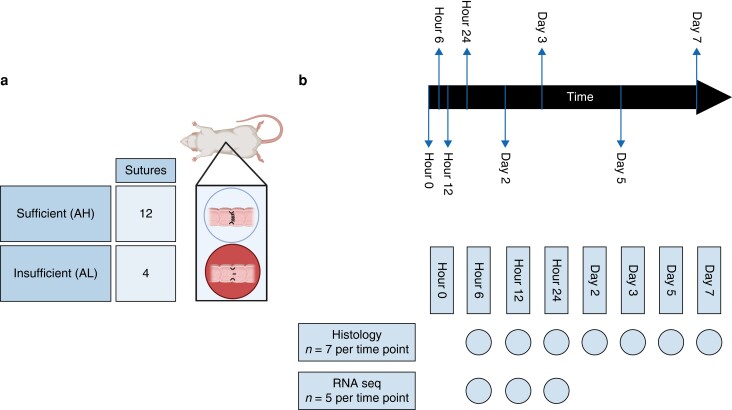
**Overview of the experiment (created with BioRender**.**com**) **a** Explanation of the used models to mimic normal anastomotic healing (AH) with the creation of a sufficient anastomosis with 12 sutures, and disturbed anastomotic healing (anastomotic leakage: AL) with the creation of an insufficient anastomosis with four sutures. **b** Timeline of experiment. The creation of the anastomosis on day 0 and killing of seven animals per group at time points 6, 12, 24 hours and 2, 3, 5, 7 days. Histologic assessment was performed on all time points using all seven animals and RNA sequencing on the first three time points using five animals.

To investigate AL and AH, rats underwent surgery and a colorectal anastomosis was created in the proximal colon. For rats that were assigned to the healing model (AH), a sufficient anastomosis with 12 sutures was created, whereas for rats assigned to the leakage model (AL), an insufficient anastomosis was created, which consisted of 4 stitches (*Fig. 1a*)^12^. Since this was an exploratory analysis with no previous data, a power calculation was not performed. Rats were operated upon on day 0. Per model, seven rats were assigned to seven different postoperative follow-up intervals, namely 6 hours, 12 hours, 24 hours, 2 days, 3 days, 5 days and 7 days. At the given follow-up time point the rats were killed, whereupon the anastomosis was resected and processed for further analysis (*Fig. 1b*). If an animal showed signs of sepsis or distress prior to their assigned time point of killing, humane termination was executed and the animal was excluded from further analysis. Excluded animals were not replaced.

### Surgical procedure introducing the anastomosis

Rats were operated on by two experienced MDs PhDs (A.C.H.M.J. and J.W.A.M.B.). The rats were anaesthetized using isoflurane and received buprenorphine as an analgesic; no antibiotic prophylaxis was used. The abdomen was opened via a 5 cm median abdominal incision. The caecum was identified and mobilized in the abdominal cavity. To protect the caecum from dehydration, it was covered with sterile saline infused gauzes. The colon was transected 2 cm distal from the caecum and an end-to-end anastomosis with full thickness stiches was created. In the AH model 12 interrupted Prolene 6/0 (Ethicon, Johnson & Johnson, Cincinnati, OH, USA) sutures were used and 4 interrupted stiches were used in the AL model. After creation of the anastomosis, the colon was repositioned in the abdominal cavity. The muscle layer was closed using a running Vicryl 4/0 (Ethicon, Johnson & Johnson, Cincinnati, OH, USA) suture, and the skin was closed with an intracutaneous running Monocryl 4/0 (Ethicon, Johnson & Johnson, Cincinnati, OH, USA) suture.

### Macroscopic assessment of our endpoints

The macroscopic endpoint was defined using the anastomotic complication score (ACS)^14^. A score between 0 and 2 is indicative of no AL whereas a score ≥3 is suggestive of AL (*Table 1*). The assessment was performed by the same researchers that performed the operation. No blinded assessment was conducted due to the visibility of the number of sutures that was used to create the anastomosis.

**Table 1:** Anastomotic complication score (ACS)^14^

Anastomotic complication score
0 = no adhesions or abnormalities
1 = adhesions to fat pad, clean anastomosis underneath
2 = adhesion to intestinal loop, abdominal wall or other organ
3 = anastomotic defect found underneath adhesion, no other abnormalities
4 = signs of possible contamination (for example small abscesses)
5 = clear anastomotic complication; spread of pus, obstruction at anastomosis, signs of peritonitis
6 = faecal peritonitis/death due to peritonitis

### Tissue isolation and preparation

After being killed, the part of the colon with the anastomosis was resected and immediately embedded, frozen in liquid nitrogen and stored at −80°C.

### Histological assessment

For histological evaluation, 5 μm sections of the anastomotic tissue were stained with standard haematoxylin and eosin (H&E) and evaluated microscopically using the modified Ehrlich–Hunt score (mEHS). The mEHS is a scoring system in which 0 = no evidence, 1 = occasional evidence, 2 = light scattering, 3 = abundant evidence and 4 = confluent cells or fibres^15^. Inflammation, granulocyte infiltration, macrophage infiltration, mucosal oedema, submucosal oedema, fibroblast activity and neo-angiogenesis were blindly assessed by an independent experienced animal pathologist and a researcher.

### RNA extraction and mRNA sequencing analysis

For RNA sequencing, five tissue samples from both the AH and AL models at time point 6 hours, 12 hours and 24 hours were used. The five animals in the AL model selected were those with the highest ACS. The frozen anastomotic tissue samples were homogenized using 1.5 ml TRI-reagent (Sigma-Aldrich Chemie BV) and a tissue homogenizer (IKA Ultra-Turrax). mRNA was then isolated according to the manufacturer’s protocols. RNA concentrations were measured using the NanoDrop 1000 spectrophotometer (Thermo Scientific) and quality was assessed using the Agilent 2200 Tapestation (Agilent technologies Netherlands BV, Amstelveen, The Netherlands), where RNA samples with an RNA Integrity Number (RIN) score of ≥7.5 were deemed of sufficient quality for subsequent analysis.

These samples were then converted into cDNA for sequencing using the KAPA mRNA Hyperprep kit (Roche Diagnostic Nederland BV, Almere, The Netherlands)^16^. Subsequently, cDNA was sequenced in a 50 bp single-ended fashion to a depth of 40 million on the Illumina HiSeq4000 at the Amsterdam UMC Core Genomics Facility. Quality control of the reads was done with FastQC (v0.11.8) and MultiQC (v1.0)^17,18^. Raw reads were aligned to the rat genome (Rnor6.0) using STAR (v2.7.7) and annotated using the Ensembl v95 annotation^19^. Postalignment processing was performed through SAMtools (v1.9), after which reads were counted using the featureCounts function found in the Subread package (v1.6.3)^20,21^.

### Statistical analyses

Statistical analyses were performed using GraphPad Prism version 9.1.0 for Windows (GraphPad Software, San Diego, CA, USA, www.graphpad.com). Normality was tested using the Kolmogorov–Smirnov test. Continuous variables are presented as mean with standard deviation (s.d.) and compared using the Student’s *t* test or Mann–Whitney *U* test as appropriate. Changes over time were compared using ANOVA or the Kruskal–Wallis test as appropriate. Differential expression (DE) analyses were performed using the Bioconductor (v3.14) package DESeq2 (v1.34.0) in the R statistical environment (v4.1.2). Altogether, transcripts of ∼24 000 genes were measured. Initial exploratory analyses were performed using principal component analysis (PCA), which is a dimension reduction method that seeks to summarize the information of all ∼24 000 genes into a reduced dimension space that carries the most variance. Initially, time-series analyses on AH rats were performed. To this end, a combination of likelihood ratio test, as well as subsequent pairwise comparisons were performed to identify genes associated with time and categorize aforementioned genes into early- or late-onset genes. Subsequent analyses focused on differences between AH rats and AL rats by performing likelihood ratio tests with an interaction between leakage status and time^22–24^. In all analyses, differentially expressed genes (DEGs) were defined as genes whose difference presented a Benjamini–Hochberg-adjusted *P* < 0.05. Gene set enrichment analysis was conducted with the fgsea package (v1.20) using the Kyoto Encyclopedia of Genes and Genomes (KEGG) database as reference. The KEGG database is a curated data set of genes in which protein products are known to function together and are hence used in gene set enrichment analyses to gain insight into the potential functional aspects of the differences in expression. Hierarchical clustering analysis was performed using DEGreport (v1.32.0) whereupon manual curation was performed afterwards. Visualizations were created in ggplot2 (v3.3.5)^25–27^.

## Results

### Macroscopic and histological assessment of rats presenting with anastomotic healing

**Fig. 2:**
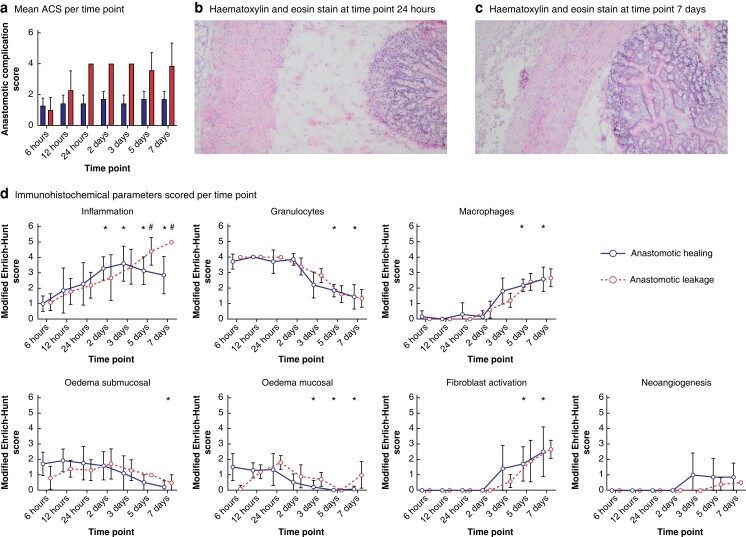
Macroscopic and histopathologic assessment of the anastomotic tissue of the healing (AH) and leakage (AL) model **a** Mean anastomotic complications score (ACS) per time point for the AH and AL model. **b** Haematoxylin and eosin (H&E) stain picture of colonic wall 24 hours (24 h) after surgery. **c** Haematoxylin and eosin (H&E) stain picture of colonic wall 7 days (7 d) after surgery. **d** Immunohistochemical parameters scored at the seven different time points using the modified Ehrlich–Hunt Score (mEHS) for the AH and AL model. *=significantly different score for AH compared with score for AH at time point 6 hours. #=significantly different score of AH compared with AL.

The population of rats comprised 98 animals (N_AL_ = 49; N_AH_ = 49). All animals in the AH model completed follow-up. The overview of animal outcomes and inclusion in analyses of the AH model is provided in *Supplementary Materials* (*Table S1*). Macroscopic evaluation of the anastomosis indicated no signs of AL, with the mean ACS ranging from 1.3 (0.49) to 1.7 (0.49) across the different days (*Fig. 2*). Next, histological assessment was performed on the anastomotic tissue to evaluate changes in the tissue over time during the healing process. The influx of inflammatory cells was observed as early as 6 hours and gradually increased over time, peaking at 2–3 days, before slightly decreasing again. Compared with 6 hours, the influx of inflammatory cells was significantly increased at time point 2 days (*P* = 0.002), 3 days (*P* = 0.003), 5 days (*P* = 0.010) and 7 days (*P* = 0.041). While neutrophils were the most common inflammatory cells present in the tissue during the first 2 days, there appeared to be a shift towards an increased macrophage presence thereafter. The decrease in neutrophils was significant at 5 days (*P* = 0.026) and 7 days (*P* = 0.003) compared with 6 hours. The increase of macrophages was significantly increased at these two time points, with *P* = 0.011 and *P* = 0.001 respectively. Oedema was evident in the submucosal and mucosal layers of the colonic wall at 6 hours and increased until 12–24 hours after surgery. After 24 hours it showed a gradual decrease which was significant at 7 days (*P* = 0.007) in the submucosa and at 3 days (*P* = 0.045), 5 days (*P* = 0.004) and 7 days (*P* = 0.008) in the mucosal layer compared with 6 hours. Collagen deposition by fibroblasts was first visible at 3 days and increased significantly at 5 days (*P* = 0.014) and 7 days (*P* = 0.002). Vascular proliferation was also first visible at 3 days. The epithelium of the colon showed almost complete healing after 7 days, but the serosa remained inflamed (*Fig. 2*), as shown in representative H&E images at 24 h and 7 d (*Fig. 2*).

### Macroscopic and histological assessment of rats that presented with disturbed anastomotic healing

In the AL model, one animal died 4 days prior to completing the follow-up at 7 days due to faecal peritonitis and was excluded from further analysis. In the *Supplementary Materials*, *Table S2* gives an overview of the animal outcomes and inclusion in the analyses of the AL model. Macroscopically, none of the animals developed AL at 6 hours after surgery with a mean ACS of 1.0 (0.82). However, at 12 hours after surgery, three animals (43 per cent) developed AL with a mean ACS of 2.3 (1.25), and at 24 hours after surgery all animals had developed AL with a mean ACS of 4.0 (0), which persisted throughout 2 and 3 days after surgery. At 5 days, six of seven rats (86 per cent) developed AL with a mean ACS of 3.6 (1.13) and at 7 days five rats (83 per cent) presented with a mean ACS of 3.9 (1.46). Altogether, 35 animals (73 per cent) presented with an ACS of >3 and thus developed macroscopic AL. Comparison of the AL animals with AH indicated a significant difference in ACS at 24 hours (*P* = 0.002), 2 days (*P* = 0.005), 3 days (*P* = 0.002) and 7 days (*P* = 0.01) after surgery, but not at 6 hours (*P* = 0.719), 12 hours (*P* = 0.194) and 5 days (*P* = 0.067) after surgery (*Fig. 2*). Histologically, animals in the AL group presented with inflammation at 6 hours after surgery, which increased until 7 days after surgery. The inflammatory response was significantly higher in the AL group compared with AH at 5 (*P* = 0.041) and 7 days (*P* = 0.003). At a cellular level, no significant difference was found in the immune cell composition, but the neutrophil to macrophage shift occurred 1 day earlier under AL conditions as opposed to AH. Oedema was evident in the submucosal layer and mucosal layer, peaking after 1 to 2 days. Collagen deposition by fibroblasts was present in both groups from time point 2 days after which it increased until time point 7 days. Vascular proliferation was present from 3 days in the AL group (*Fig. 2*).

### Anastomotic healing associated with time-associated oscillation of cytokine and signalling gene expression

**Fig. 3:**
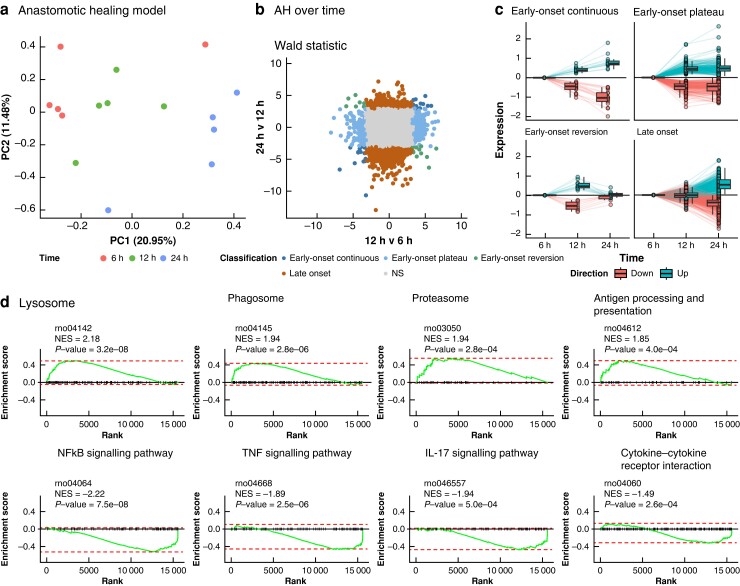

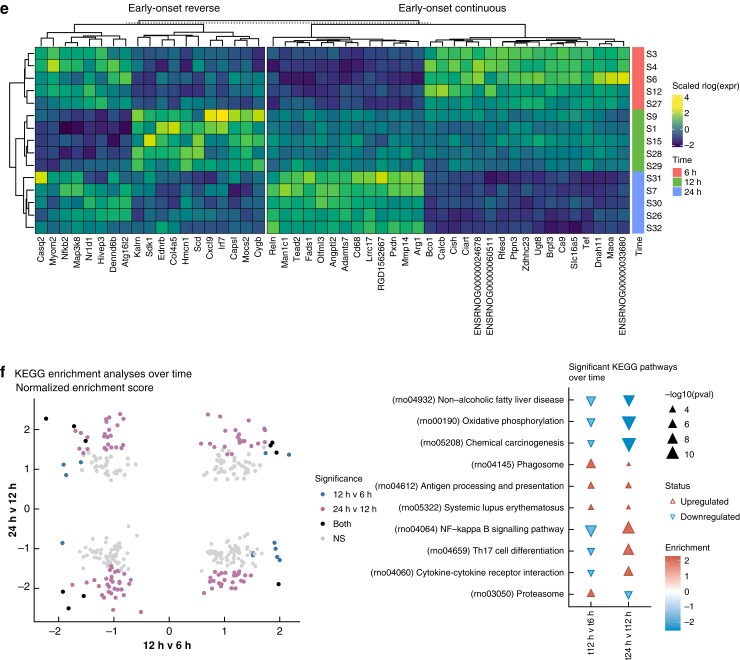
Transcriptomic analyses undergoing anastomotic healing (AH) (continued on next page)

To characterize the healing process at a molecular level, the gene expression of animals at 6, 12 and 24 hours after surgery was assessed. Principal component analysis (PCA) showed reasonable clustering by time point, indicating relatively consistent transcriptome-wide differences over time (*Fig. 3*). Through pairwise comparative analyses, 1350 DEGs were identified that were significantly associated with time (*Fig. 3b*), which were categorized as either early onset (N_DEGs_ = 431) or late onset (N_DEGs_ = 919), depending on whether they were significantly different between 6 and 12, or between 12 and 24 hours respectively (*Fig. 3*). Through gene set enrichment analyses of the differences between 6 and 12 hours, multiple KEGG gene sets associated with innate immune response as well as cell adhesion and coagulation were found to be differentially expressed. While genes associated with the lysosome (rno04142), phagosome (rno04145), proteasome (rno03050), and antigen processing and presentation (rno04612) were upregulated, and genes involved in inflammatory signalling pathways, such as the nuclear factor kappa B (NFκB) signalling pathway (rno04064), the tumour necrosis factor (TNF) signalling pathway (rno04668), the interleukin (IL)-17 signalling pathway (rno04657) and the cytokine–cytokine receptor interaction (rno04060) were all downregulated (*Fig. 3*). Subcategorizing the early-onset DEGs based on their expression between 12 and 24 hours revealed that the majority of the early-onset DEGs (N_DEGs_ = 382) would seemingly plateau in their expression, presenting no significant difference between 12 and 24 hours (‘early-onset plateau’) (*Fig. 3*). By contrast, a smaller subset of early-onset DEGs would continuously increase or decrease their expression (‘early-onset continuous’) (N_DEGs_ = 30), or would change the direction of effect between 12 and 24 hours (‘early-onset reverse’) (N_DEGs_ = 19) (*Fig. 3*). Such an early-onset reversing pattern was observed for the aforementioned inflammatory signalling pathways, which would be upregulated between 12 and 24 hours (*Fig. 3*). Taken together, it appears that anastomotic healing is characterized initially by an increasing expression of genes encoding proteins involved in clearance and antigen presentation. By contrast, genes encoding cytokine and signalling molecules present an initial downregulation between 6 and 12 hours followed by upregulation between 12 and 24 hours.

**a** Principal component analysis of the animals coloured by time (hours) after surgery. **b** Scatterplot of the effect sizes (Wald statistic) in gene expression, between 6 and 12 hours and 12 and 24 hours on the *x*- and *y*-axis respectively. Grey dots represent genes that were found to be non-significant in either of the comparisons (‘NS’), dark blue dots represent genes that present continuous change between 6 and 12 as well as between 12 and 24 hours (‘early-onset continuous’), light blue dots represent genes that present an initial change between 6 and 12 hours whereupon they plateau between 12 and 24 hours (‘early-onset plateau’), green dots represent genes that present an initial change between 6 and 12 hours whereupon they switch direction between 12 and 24 hours (‘early-onset reversion’), and orange dots represent genes that present a change only between 12 and 24 hours (‘late onset’). **c** Line and boxplot representing the expression relative to 6 hours of the significantly differentially expressed genes facetted by the aforementioned classifications. Blue and red indicate up- and downregulation respectively. **d** Barcode plots representing selected gene sets that were found to be up- and downregulated with the *x*- and *y*-axis representing the rank and enrichment score. Annotations at the top are the name of the gene set, the KEGG identifier, the normalized enrichment score (‘NES’), and the *P* value. **e** Heatmap representing the scaled gene expression of the genes identified as early-onset continuous or early-onset reversion. **f** Left: scatterplot of the effect sizes (normalized enrichment score) of the KEGG gene sets, when comparing 6 and 12 hours and 12 and 24 hours on the *x*- and *y*-axis respectively. Grey dots represent KEGG gene sets that were found to be non-significant in either of the comparisons (‘NS’), blue dots represent gene sets that present a significant difference only between 6 and 12, lilac dots represent gene sets that present a significant difference only between 12 and 24 hours, and black dots represent gene sets that are significant in both comparisons. Right: a heatmap showing a more granular effect size in colour intensity and direction of the arrow, as well as the significance in size. IL, interleukin; KEGG, Kyoto Encyclopedia of Genes and Genomes; NFKB, nuclear factor kappa B; TNF, tumour necrosis factor.

### Disturbed anastomotic healing is characterized by genes associated with T cell activity at 6 and 24 hours after surgery

**Fig. 4:**
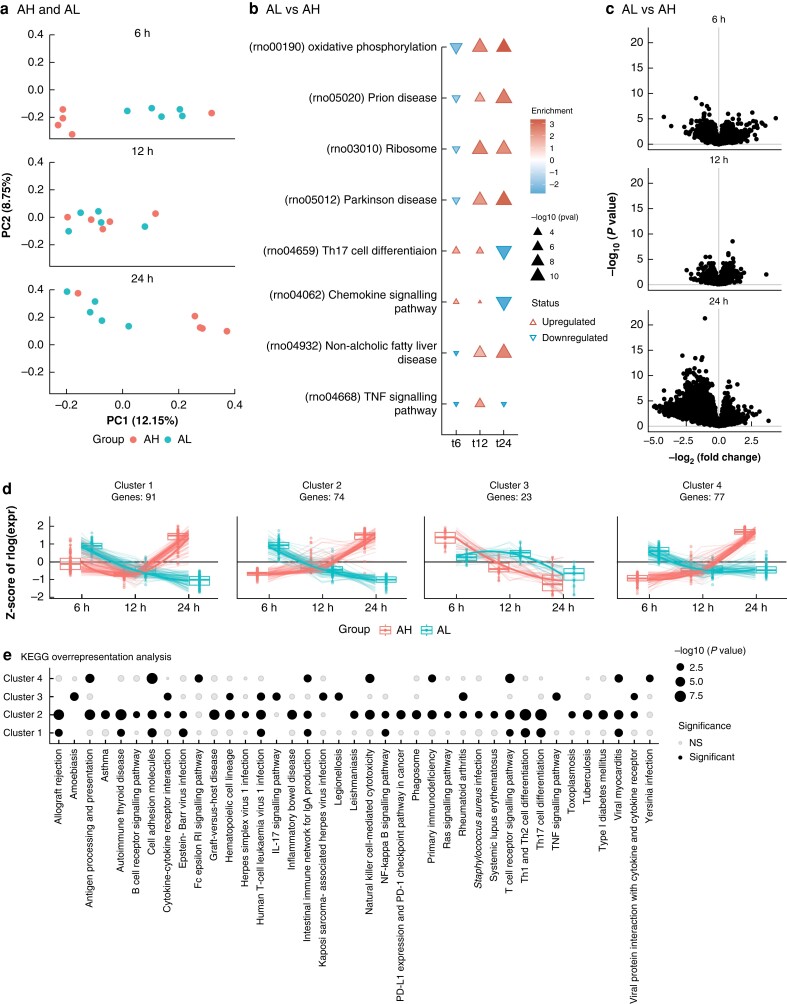
Transcriptomic differences between anastomotic healing (AH) and anastomotic leakage (AL) **a** Principal component analysis of AH and AL samples at all 3 time points, coloured by AL or AH status. **b** A heatmap representing the effect size of the significantly associated gene sets that present a different temporal behaviour when comparing AL with AH. The direction and colour of the arrow indicates the direction of effect when comparing AL with AH, while the size is proportional to the statistical significance in −log_10_(*P* value). **c** Volcano plots representing the mean difference in expression in log_2_(fold-change) on the *x*-axis and the statistical significance in −log_10_(*P* value) on the *y*-axis when comparing AL with AH for all measured genes. **d** Z-scored expression of the 265 genes categorized into 4 different classes through divisive hierarchical clustering annotated by the number of genes per class and coloured by the group. **e** Dotplot comparing the overrepresented gene sets across the different gene set clusters. The size of the dots represents statistical significance in −log_10_(*P* value) with black dots being statistically significant, whereas grey dots are non-significant.

Including the RNA sequencing results of the AL model animals at 6, 12, and 24 hours after surgery into a PC analysis indicated that AH or AL status was visibly associated with PC1 and time with PC2, suggesting that the largest differences are time associated. Notably, the separation between AH and AL was most visible at 6 and 24 hours. By contrast, samples obtained at 12 hours presented limited to no status-associated clustering (*Fig. 4*). Through a log-likelihood ratio test, the interaction between time and leakage was investigated, which yielded 336 DEGs that behaved transcriptionally different between AH and AL over time. Gene set enrichment analyses indicated significant enrichment of oxidative phosphorylation (rno00190), Th17 cell differentiation (rno04659), and the antigen processing and presentation (rno04612) gene set at every time point. Where the genes associated with oxidative phosphorylation presented lower expression in AL *versus* AH at 6 hours, the gene expression would be higher at 12 and 24 hours (*Fig. 4*). Notably, the inverse was true for antigen processing and presentation, and while Th17 cell differentiation was lower at both 6 and 12 hours, it would become higher at 24 hours. Overall, a general lower expression of genes was seen when comparing AL with AH at 24 hours (*Fig. 4*), implicating a general downregulation of genes under AL conditions. To better understand the processes underlying the difference between AH and AL over time, the DEGs were clustered using a divisive hierarchical clustering approach based on their expression profile. Of the 336 DEGs, 265 genes were categorized into four clusters (*Fig. 4*). Cluster 1 (N_DEGs_ = 91) and 2 (N_DEGs_ = 74) consisted of genes that would be significantly higher in their expression under AL conditions relative to AH and would present a continuous downregulation over time. By contrast, under AH conditions the cluster 1 and 2 genes would present a general upregulation between 12 and 24 hours. Notably, cluster 1 was overrepresented for multiple T cell-associated gene sets, including Th1, Th2 (rno04658) and Th17 cell differentiation (rno04659), as well as T cell receptor signalling (rno04660), suggesting that T cell-associated processes appear to be consistently downregulated over time under AL conditions, as opposed to the AH-associated upregulation (*Fig. 4*). Genes from cluster 3 (N_DEGs_ = 23) at 6 hours were lower in AL relative to AH and subsequently exhibited a late-onset downregulation under AL conditions, whereas under AH conditions, an early-onset continuous downregulation was expected. Overrepresentation of cluster 3-associated genes was found for the IL-17 signalling pathway (rno04657) and TNF signalling pathway (rno04668) in particular, which appear to be more gradually downregulated in the AL condition as opposed to the AH condition. Finally, the genes in cluster 4 (N_DEGs_ = 77) present a similar downregulation as clusters 1 and 2 under AL conditions, but appear to plateau between 12 and 24 hours. Under AH conditions, such genes would have been consistently upregulated. Genes in cluster 4 are overrepresented for cell adhesion molecules (rno04514), T cell receptor signalling pathway (rno04660), and natural killer cell-mediated cytotoxicity (rno04650). Taken together, there appears to be an overall downregulation in the number of genes over time in the AL group, which is largely associated with T cell-associated gene sets.

## Discussion

In this longitudinal study, the fundamental processes over time underlying AH and disturbed anastomotic healing were investigated in a rat model. Through a multimodal approach, the anastomosis was assessed at macroscopic, histopathologic and transcriptomic level, providing insights into the morphological, cellular and intrinsic molecular changes in the anastomotic tissue. None of the animals in the AH model developed macroscopic AL. In the AL model, at time point 12 h the first animals showed signs of AL and at time point 24 hours, 2 days and 3 days all animals developed AL macroscopically.

The modified Ehrlich–Hunt score was used to contextualize our observations within the classically defined three-stage process of wound healing in skin. The intestinal wound healing showed similar phases to skin wound healing, namely inflammation, proliferation and remodelling. In the inflammation phase of the skin, neutrophils and macrophages predominate within 2–3 days. In the AH model, inflammation was already visible at 6 hours and was most abundant at 2–3 days after which it decreased; neutrophils were the dominant cell type until 2 days and at 3 days, macrophages became more dominant. A decrease of neutrophils and increase of macrophages indicate in the skin the transition from an inflammatory to proliferative phase^28^. The presence of macrophages increased until day 7 in our model. Macrophages are drivers of proliferation in the skin, as they both promote fibroblast activation and neovascularization. In the skin, fibroblasts become dominant after 4 days^28,29^. Fibroblast activation in the colonic tissue was first observed at 3 days, the same as the first signs of neoangiogenesis. To assess these specific changes in the proliferative phase and later on remodelling phase, H&E staining is not the preferred staining. Preferably, additional staining would have been performed for fibroblasts/collagen formation and vascularization. However, the aim was to generate a general assessment of the early phases of wound healing in the intestine after creation of an anastomosis using H&E staining and mEHS assessment and to use the other anastomotic tissue for assessing molecular changes in the anastomosis via RNA sequencing.

Analysing the observed transcriptomic processes, a multitude of DEGs linked to pathways that are predominantly involved in inflammatory and wound healing processes were found. Unlike the histopathological outcomes, these processes cannot be strictly classified within a single stage of the three phases of wound healing, with particular inflammatory and proliferation processes co-occurring largely simultaneously. The authors acknowledge that they are not looking at a human, nor are they looking at a cutaneous wound. Furthermore, wound healing in the intestine is far more rapid than in the skin, therefore distinction between the classical phases may be less clear in intestinal wound healing than in the skin^30^.

The observation that lysosome- and phagosome-related pathways are upregulated, while typical inflammatory signalling pathways were downregulated and then subsequently upregulated, entices us to speculate that a dynamic process occurs throughout the first day after surgery. This process appears to be altered in disturbed healing conditions, which expectedly shows a higher expression of multiple immune and inflammatory pathways, but then presents a surprisingly consistent decrease in gene expression over time. Notably, a significant association of the Th17 cell differentiation gene set was observed. The role of Th17 cells is mainly investigated in inflammatory bowel disease animal models, where these specific cells are involved in multiple processes after mucosal damage^31^. Th17 cells play a role in host defence against microbiota at the mucosal site by attracting and activating other immune cells, but also play a role in epithelial proliferation and tissue regeneration by releasing cytokines, such as interleukin (IL)-17, IL-22 and tumour necrosis factor-α (TNF-α). These mechanisms need to be regulated, otherwise inflammation and wound healing can become pathogenic^31–33^. IL-17 is the hallmark cytokine of Th17 cells, which seems to have both promoting and inhibiting effects on wound healing^31^. Interestingly, the IL-17 signalling pathway was also significantly different between AH and AL in our study, suggesting that Th17 cells and Th17 cell-related cytokines might play an important role in the activation and maintenance/inhibition of the inflammatory response during the first phase of wound repair.

Besides the multiple T cell-associated gene sets, several other gene sets are differentially expressed between AH and AL. While most are involved in the immune response, some gene sets are associated with cell metabolism, cell adhesion molecules and several gene sets which cannot be directly placed in the known processes that are described in AH and AL, such as prion disease and Parkinson disease pathways. This diverse group of gene sets also shows that on a molecular level, the healing and disturbed healing process is multifactorial and researchers should look further than the expected processes associated with immune response and cell proliferation to elucidate the underlying mechanisms of colorectal anastomotic healing and leakage.

There are several limitations to this study. First, the use of a leakage model in which leakage is induced using an inadequate anastomotic construction. An insufficient anastomosis will never be intentionally created by a surgeon, however technical failure of the anastomosis is not uncommon in daily clinical practice. It is suggested that there are two different types of AL, namely early AL and late AL. The risk factors of early AL are mostly surgery-related risk factors and for late AL risk factors are mostly patient related. Surgical-related risk factors represent surgical difficulty which could result in technical failure of the anastomosis, whereas patient-related risk factors represent the physical status of the patient influencing the quality and rapidity of the healing process^34^. Therefore, the used leakage model is regarded as a suitable model to investigate AL in animals. Second, in this model, full thickness stitches were used not taking into account the alignment of different bowel layers. According to a past study, this model represents secondary wound healing, as full thickness stitches result in non-alignment of bowel layers which leads to an intestinal wound filled with necrosis, debris and bacteria, which the body must clear before the intestinal layers can heal, whereas primary wound healing, where bowel layers are aligned, would lead to less necrosis, granulation tissue and abscesses resulting in faster and better wound healing than secondary wound healing^35^. The best technique to perform an intestinal anastomosis is still a topic of debate and multiple studies have investigated multiple techniques without a clear conclusion. Nowadays, most anastomoses are performed using stapling devices disregarding the alignment of bowel wall layers^36^. Nevertheless, taking into account the differences between primary and secondary wound healing is important and further research is needed into which manner of healing results in the best healed anastomosis together with more research into the role of the individual bowel layers in the healing process. Third, as we have focused on early AL with RNA sequencing, we have not taken into account other factors contributing to the multifactorial process of anastomotic healing and leakage, for example the role of the microbiome. Multiple animal studies have provided evidence that bacteria could play a role in the pathogenesis of AL, for example collagenolytic *Enterococcus faecalis*^37^. As there is still a lacuna in the knowledge of the processes in normal and disturbed healing on a tissue level, one of the aims was to elucidate this, before combining multiple factors. Finally, some extremely interesting observations, such as the decrease in T cell gene expression over time, have not yet been validated. Validation of these findings, using for example a TH17 knock down model, could be of great value and hopefully support our conclusions in the future.

Taken together, a histologically dynamic process in intestinal wound healing after creation of an anastomosis was observed that is in line with the sequential order of skin wound healing, namely inflammation, proliferation and remodelling. Furthermore, inflammation was significantly increased at 5 and 7 days in the disturbed healing model compared with the healing model. Large-scale transcriptomic differences were observed between AH and AL at 6 hours, whereas macroscopic differences were only observable from 12 hours onwards. These observations could therefore be harnessed as potential early-onset biomarkers of AL, which in turn could be used as the first step to truly understand the disrupted healing processes underlying AL.

## Supplementary Material

zrad099_Supplementary_Data

## Data Availability

Original data available on request.
