# Supplementary material for: Histology and transcriptome insights into the early processes of intestinal anastomotic healing: a rat model
Source: BJS Open. 2023 Oct 19;7(5):zrad099. doi: 10.1093/bjsopen/zrad099 (PMC10586197; doi:10.1093/bjsopen/zrad099)
Supplement: zrad099_Supplementary_Data [file zrad099_supplementary_data.docx]

**Histology and transcriptome insights into the early processes of intestinal anastomotic healing: a rat model**

Claire P.M. van Helsdingen^1,2,3,4^, Aurelia C.L. Wildeboer^1,5^, Konstantina Zafeiropoulou^1,2,3^, Audrey C.H.M. Jongen^6^, Joanna W.A.M. Bosmans^6^, Camille Gallé^7^, Theodorus B.M. Hakvoort^2,3^, Marion J.J. Gijbels^8,9,10,11,12^, Wouter J. de Jonge^2,3,13^, Nicole D. Bouvy^6,9^, Andrew Y.F. Li Yim^1,2,3,4,14^ and Joep P.M. Derikx^1,3,4^

^1^Emma Children’s Hospital, Amsterdam UMC, location University of Amsterdam, Paediatric Surgery, Meibergdreef 9, Amsterdam, The Netherlands

^2^Amsterdan UMC, location University of Amsterdam, Tytgat Institute for Liver and Intestinal Research, Meibergdreef 9, Amsterdam, The Netherlands

^3^Amsterdam Gastroenterology Endocrinology Metabolism, Amsterdam, The Netherlands

^4^Amsterdam Reproduction and Development, Amsterdam, The Netherlands

^5^GROW, School for Oncology and Developmental Biology, Maastricht University, Maastricht, 6229 ER, The Netherlands

^6^Department of Surgery, Maastricht University Medical Center, Maastricht, 6200 MD, The Netherlands

^7^Department of General Surgery, Maastricht University, Maastricht, The Netherlands

^8^Department of Pathology, Maastricht University Medical Center, Maastricht, The Netherlands

**^9^**NUTRIM School of Nutrition and Translational Research in Metabolism, Maastricht University Medical Center, Maastricht, 6229 HX, The Netherlands

^10^Amsterdam UMC, location University of Amsterdam, Department of Medical Biochemistry, Experimental Vascular Biology, Meibergdreef 9, Amsterdam, The Netherlands

^11^Amsterdam Infection and Immunity, Amsterdam, The Netherlands

^12^Amsterdam Cardiovascular Sciences, Amsterdam, The Netherlands

^13^Department of Surgery, University of Bonn, Bonn, Germany

^14^Amsterdam UMC, location University of Amsterdam, Genome Diagnostics Laboratory, Department of Human Genetics, Meibergdreef 9, Amsterdam, The Netherlands

C.P.M.v.H. and A.C.L.W. are shared first authors.

Corresponding author: Claire P. M. van Helsdingen, Emma Children’s Hospital, Amsterdam UMC, location University of Amsterdam, Paediatric Surgery, Meibergdreef 9, 1105 AZ, Amsterdam, The Netherlands (e-mail: [c.p.vanhelsdingen@amsterdamumc.nl](mailto:c.p.vanhelsdingen@amsterdamumc.nl)). ORCID:0000-0001-6508-0214.

**Supplementary Materials - Index**

| **Supplementary Figures and Tables** |  |
| --- | --- |
| Supplementary Table 1. Table of animal outcomes and inclusion in analyses of the anastomotic healing model | *Page 4* |
| Supplementary Table 2. Table of animal outcomes and inclusion in analyses of the anastomotic leakage model | *Page 4* |

**Supplementary Figures and Tables**

**Supplementary Table 1. Table of animal outcomes and inclusion in analyses of the anastomotic healing model**

|  | 6 hours | 12 hours | 24 hours | 2 days | 3 days | 5 days | 7 days |
| --- | --- | --- | --- | --- | --- | --- | --- |
| Animals assigned to group | 7 | 7 | 7 | 7 | 7 | 7 | 7 |
| Animals reached endpoint | 7 | 7 | 7 | 7 | 7 | 7 | 7 |
| Animals used for macroscopic assessment | 7 | 7 | 7 | 7 | 7 | 7 | 7 |
| Animals that developed anastomotic leakage macroscopically | 0 | 0 | 0 | 0 | 0 | 0 | 0 |
| Animals used for microscopic assessment | 7 | 7 | 7 | 7 | 7 | 7 | 7 |
| Animals used for RNA sequencing | 5 | 5 | 5 | 0 | 0 | 0 | 0 |

**Supplementary Table 2. Table of animal outcomes and inclusion in analyses of the anastomotic leakage model.**

|  | 6 hours | 12 hours | 24 hours | 2 days | 3 days | 5 days | 7 days |
| --- | --- | --- | --- | --- | --- | --- | --- |
| Animals assigned to group | 7 | 7 | 7 | 7 | 7 | 7 | 7 |
| Animals reached endpoint | 7 | 7 | 7 | 7 | 7 | 7 | 6 |
| Animals used for macroscopic assessment | 7 | 7 | 7 | 7 | 7 | 7 | 6 |
| Animals that developed anastomotic leakage macroscopically | 0 | 3 | 7 | 7 | 7 | 6 | 5 |
| Animals used for microscopic assessment | 7 | 7 | 7 | 7 | 7 | 7 | 6 |
| Animals used for RNA sequencing | 5 | 5 | 5 | 0 | 0 | 0 | 0 |
